# FAM83B is a novel biomarker for diagnosis and prognosis of lung squamous cell carcinoma

**DOI:** 10.3892/ijo.2015.2817

**Published:** 2015-01-07

**Authors:** NAOYUKI OKABE, JUNJI EZAKI, TAKUMI YAMAURA, SATOSHI MUTO, JUN OSUGI, HIROSUMI TAMURA, JUN-ICHI IMAI, EMI ITO, YUKA YANAGISAWA, REIKO HONMA, MITSUKAZU GOTOH, SHINYA WATANABE, SATOSHI WAGURI, HIROYUKI SUZUKI

**Affiliations:** 1 Department of Regenerative Surgery, Fukushima Medical University, School of Medicine, Fukushima 960-1295, Japan; 2 Medical-Industrial Translational Research Center, Fukushima Medical University, School of Medicine, Fukushima 960-1295, Japan; 3 Nippon Gene Co., Ltd., Chiyoda, Tokyo 101-0054, Japan

**Keywords:** FAM83B, squamous cell carcinoma, diagnostic marker, prognostic marker, non-small cell lung cancer

## Abstract

Personalized therapy for non-small cell lung cancer (NSCLC), particularly lung adenocarcinoma, has recently been significantly improved by the discovery of various molecular targets. However, this has not been the case for lung squamous cell carcinoma (SCC). In the present study, we identified the family with sequence similarity 83, member B (FAM83B) as a candidate marker for SCC through a comprehensive gene expression analysis and examined its correlations with various clinicopathological factors. The subjects of this study consisted of 215 patients with NSCLC who underwent complete resection from 2005 to 2011 at the Fukushima Medical University Hospital (Fukushima, Japan). They included 102 patients with adenocarcinoma and 113 with SCC. FAM83B expression was first examined in some of the samples by gene expression analysis and western blotting, and then all clinical specimens were evaluated by immunohistochemistry (IHC). The relationship between the quantitative values for IHC and clinicopathological factors was statistically analyzed. The results showed that FAM83B mRNA expression was significantly higher in SCC than in normal lung or adenocarcinoma (P<0.0001). Immunoblot analysis also confirmed this trend. Specimens containing >10% positive area for FAM83B were judged as ‘positive’; 94.3% (107/113) of SCC and 14.7% (15/102) of adenocarcinoma were positive. Patients were divided into two subgroups according to expression (54 high-expression and 53 low-expression patients); the high-expression group was associated with a better disease-free survival (DFS) rate (P=0.042, log-rank test). In conclusion, FAM83B may be a reliable diagnostic and prognostic biomarker for SCC. Detailed analyses of FAM83B function in lung cancer are required to understand how its expression is associated with better prognosis in SCC.

## Introduction

Lung cancer is a major cause of cancer-related mortality worldwide, accounting for 18% (1.4 million) of cancer deaths in 2008 (1). It is traditionally classified into two major subtypes, small cell lung cancer and non-small cell lung cancer (NSCLC), the latter of which covers ~85% of newly diagnosed lung cancers, and is further subdivided into two major histological subtypes, adenocarcinoma and squamous cell carcinoma (SCC), which account for ~38 and 20% of all lung cancers, respectively (2). Although historically these histological subtypes did not significantly affect treatment decisions (3), recent advances in NSCLC chemotherapy have introduced treatment options that are subtype-dependent. For example, a folate antimetabolite, pemetrexed, has been used in first-line (4), second-line (5), and maintenance (6) settings for patients with non-SCC. Bevacizumab, an angiogenesis inhibitor, is considered inadequate for SCC because it increases the risk of fatal hemoptysis and is less effective (7,8). In addition, discovery of biomarkers such as recurrent mutations in the epidermal growth factor receptor (EGFR) kinase and a fusion gene of EML4-anaplastic lymphoma kinase has led to a marked change in lung adenocarcinoma treatment (9–12). However, these mutations occur only in adenocarcinoma patients who have never smoked, but are not present in SCC cases that are invariably associated with tobacco smoking (13). In fact, targeted agents developed for lung adenocarcinoma have been largely ineffective against SCC (14), therefore SCC is still treated by conventional platinum-based chemotherapy with little improvement (3).

Recently, some studies have reported several genetic changes related to SCC, such as amplification of *TP63*, *PIK3CA*, *PDGFRA*, *SOX2*, or *FGFR1* and mutations in *TP53*, *EGFR*, *PIK3CA*, *NRE2L2*, *PTEN*, and *DDR2* (15,16). However, they have not been effective in the clinical setting thus far. For this reason, many researchers are exploring driver mutations as well as targeted agents for SCC through gene expression profiling and sequencing studies (16,17). Therefore, we carried out a comprehensive gene expression analysis to identify genes that are specifically and highly expressed in lung SCC, and detected the family with sequence similarity 83, member B (*FAM83B*) gene. FAM83B has been reported as an important intermediary in EGFR/RAS signaling, and is highly expressed at the mRNA level in several cancers, including breast, cervix, bladder, lung, testis thyroid, and ovary cancer (18). However, detailed examination of its protein expression and association with clinicopathological factors in SCC patients has not been previously undertaken. Therefore, we conducted western blotting and immunohistochemical analyses and found that FAM83B protein was also increased in lung SCC compared with lung adenocarcinoma or adjacent normal tissues, and that high-expression levels of FAM83B were associated with a high disease-free survival (DFS) rate.

## Materials and methods

### Ethics statement

This study was approved by the Ethics Committee of Fukushima Medical University (Fukushima, Japan) (approval no. 1713). Written informed consent was obtained from all participants involved. We obtained ethics approval from the Ethics Committees at all Institutions where samples were analyzed.

### Case selection

This study was conducted in a cohort of patients with NSCLC who underwent pulmonary resection at Fukushima Medical University Hospital between 2005 and 2011. The tumor samples of 215 patients (SCC 113 cases, adenocarcinoma 102 cases) were examined for FAM83B expression. Tumor samples were selected from patients who fulfilled all of the following criteria: i) patients suffering from primary NSCLC with confirmed stage (T1–T3, pN0-pN2, and pM0); ii) patients who underwent curative surgery but did not receive any preoperative treatment; and iii) patients whose clinical follow-up data were available. Follow-up information of at least 5 years was available for this study. Use of all clinical materials was approved by the Institutional Ethics Committee in Fukushima Medical University. Formalin-fixed, paraffin-embedded samples from all cases were used for immunohistochemistry (IHC). For the comprehensive gene expression analysis, 64 normal lung samples, 60 cases of adenocarcinoma, and 20 cases of SCC were used. For western blotting, three normal lung samples, 5 cases of adenocarcinoma, and 5 cases of SCC were used.

### Comprehensive gene expression analysis

A small fraction (7×7 mm) of each surgical specimen was excised and frozen in liquid nitrogen. Frozen samples were processed for total RNA extraction using ISOGEN (Nippon Gene Co., Ltd., Tokyo, Japan) and for purification of poly(A)^+^+RNA using a MicroPoly(A)Purist kit (Ambion, Austin, TX, USA). Human common reference RNA was prepared by mixing equal amounts of poly(A)+RNA extracted from 22 human cancer cell lines (A431, A549, AKI, HBL-100, HeLa, HepG2, HL60, IMR-32, Jurket, K562, KP4, MKN7, NK-92, Raji, RD, Saos-2, SK-N-MC, SW-13, T24, U251, U937, and Y79) to reduce cell type-specific bias in expression (19).

Synthetic polynucleotides (80-mers) representing 31,797 species of human transcripts (MicroDiagnostic, Tokyo, Japan) were arrayed using a custom arrayer. SuperScript II (Invitrogen Life Technologies, Carlsbad, CA, USA) and Cyanine 5-dUTP (Perkin-Elmer, Boston, MA, USA) was used to synthesize labeled cDNA from 2 μg sample RNA, while Cyanine 3-dUTP (Perkin-Elmer)-labeled cDNA was synthesized from 2 μg reference RNA. Hybridization was performed with a Labeling and Hybridization kit (MicroDiagnostic). Signals were measured with a GenePix 4000B Scanner (Axon Instruments, Inc., Union city, CA, USA) and then processed into primary expression ratios (ratio of the cyanine-5 intensity of each sample to the cyanine-3 intensity of the human common reference RNA). Each ratio was normalized by multiplication with the normalization factors using GenePix Pro 3.0 software (Axon Instruments, Inc.). The primary expression ratios were converted into log_2_ values (designated log ratios). We assigned an expression ratio of 1 (log ratio of 0) for spots that exhibited fluorescence intensities under the detection limits, and we included these in the signal calculation of the mean averages. The data were processed using Microsoft Excel software (Microsoft, Bellevue, WA, USA) and MDI gene expression analysis software package (MicroDiagnostic). Data corresponding to FAM83B were extracted, and statistical analysis of the Kruskal-Wallis test was performed using GraphPad Prism ver. 6.0 (GraphPad Software, Inc., San Diego, CA, USA).

### Immunoblotanalysis

Frozen tissues from tumor and non-tumor regions were homogenized with RIPA buffer [150 mM sodium chloride, 1% NP-40, 1% sodium deoxycholate, 0.1% sodium dodecyl sulfate (SDS), 25 mM Tris-HCl (pH 7.6)] containing protease inhibitor cocktail (Roche Diagnostics, Indianapolis, IN, USA) using a glass homogenizer (Wheaton Dounce Tissue Grinder) on ice. The homogenate were centrifuged at 10,000 × g at 4°C for 20 min to remove debris, and the supernatants were mixed with an equal volume of a 20 mM Tris-HCl (pH 6.8) buffer containing 2% SDS, 12% glucose, 2% 2-mercaptoethanol, 0.002% PBS, and BPB. The proteins contained in the supernatants were separated by SDS-PAGE using 5–20% gradient polyacrylamide gels (SuperSep Ace 5–20%; Wako Pure Chemical Industries, Ltd., Osaka, Japan) according to the Laemmli method (20). Separated proteins were transferred to polyvinylidene difluoride membranes (Millipore, Billerica, MA, USA), according to the method of Towbin *et al* (21). The membranes were blocked with 5% skim milk in T-PBS (0.137 M NaCl, 2.6 mM KCl, 1.8 mM KH_2_PO_4_, 8.1 mM Na_2_HPO_4_·12H_2_O, and 0.005% Tween-20) and incubated with primary antibodies overnight at 4°C. The membranes were incubated with anti-FAM83B (1:2,000, HPA031464; Atlas Antibodies AB, Stockholm, Sweden) or anti-GAPDH (1:2,500, no. 2118; Cell Signaling Technology, Inc., Danvers, MA, USA) as primary antibodies, and then with horseradish peroxidase-conjugated goat anti-rabbit immunoglobulin G antibody (1:10,000; GE Healthcare Life Sciences, Tokyo, Japan) as a secondary antibody. The signals were detected by ImageQuant LAS 4000 using Prime Western Blotting Detection Reagent (both from GE Healthcare Life Sciences).

### IHC and quantitative analysis

Tissue specimens were fixed in formalin and embedded in paraffin. Sections were autoclaved in 0.01 M citrate buffer (pH 6.0) for antigen retrieval. After blocking in 5% skim milk, sections were incubated with a rabbit polyclonal anti-FAM83B antibody (HPA031464; Atlas Antibodies AB) at a dilution of 1:100 at 4°C overnight. They were further incubated for 20 min at room temperature with a biotinylated goat anti-rabbit IgG (1:400 dilution, Vectastain Elite ABC kit; Vector Laboratories, Inc., Burlingame, CA, USA), and then with avidin-biotin-HRP regent (1:200 dilution; Vectastain Elite ABC kit; Vector Laboratories, Inc.) for 30 min at room temperature. They were observed under a microscope (BX50; Olympus, Tokyo, Japan) and positivity was judged when >10% of the area was occupied with positive cells.

For quantitation of staining intensity, tissue sections were immunohistochemically stained without nuclear counter staining. For each specimen, five regions of 680×860 μm each were randomly selected, and the images were captured with a microscope (BX51) equipped with a 20× objective lens (UPlanSApo) and a CCD camera (DP71) (all from Olympus). Images with no tissues sections were also acquired as a background signal. All images were converted to 256-level gray scale images and then inverted using ImageJ software (National Institutes of Health, Bethesda, MD, USA). Mean intensity values were measured only in the tumor regions, from which the background value was subtracted. According to the values, patients were divided into two groups; values with equal or higher than median were classified into a ‘high-expression group’, while those less than the median into a ‘low-expression group’.

### Statistical analysis

Associations of FAM83B expression levels with clinical characteristics were evaluated using Pearson’s χ^2^ test. DFS and overall survival (OS) in patients with completely resected lung cancers were analyzed. DFS was measured from the time of surgery to initial tumor relapse (local recurrence or distant). OS was calculated from the time of surgery to death at last follow-up date, and 95% confidence interval was evaluated by survival analysis using the Kaplan-Meier method. Survival outcomes for the high- versus the low-expression group were compared using the log-rank test. Statistical significance was set at P<0.05 for all analyses. Multivariate analysis was performed using Cox regression analysis with the following pre-specified variables: gender, pathological stage, smoking, and FAM83B status. All statistical analyses were performed using SPSS version 20.0 software (SPSS, Inc., Chicago, IL, USA). P<0.05 was considered statistically significant.

## Results

### FAM83B expression in lung cancer and normal tissue

**Figure 1:**
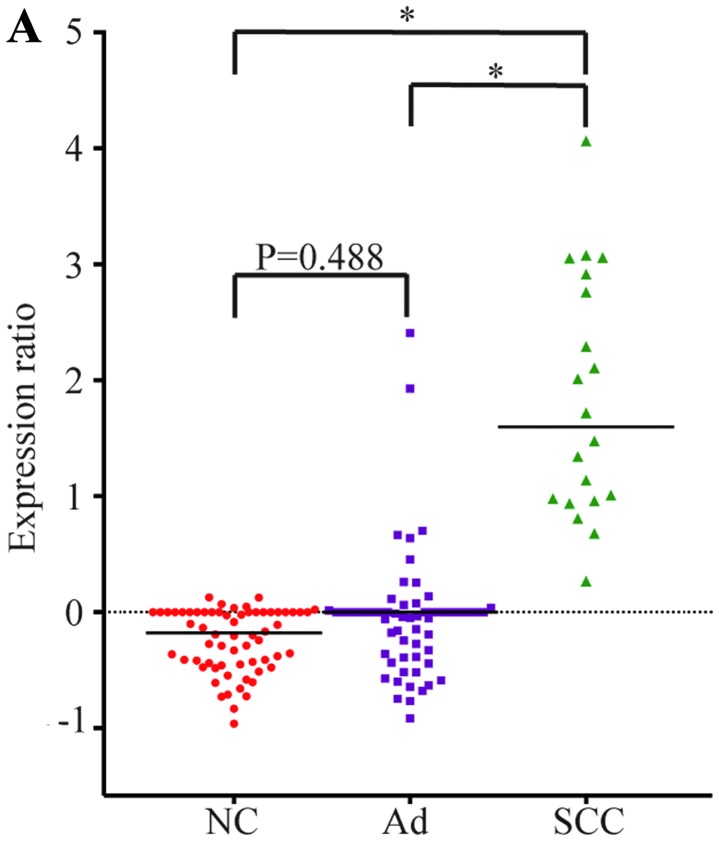

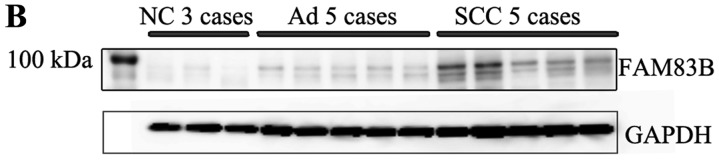

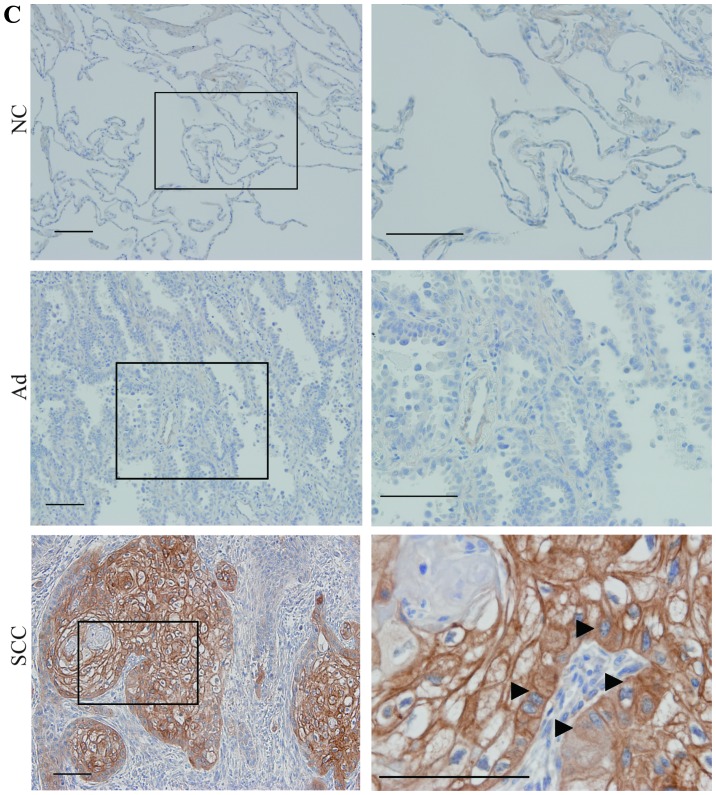
The family with sequence similarity 83 member B (FAM83B) expression in lung cancer and adjacent normal tissue. (A) Expression ratios for FAM83B mRNA in clinical samples of adenocarcinoma (Ad), squamous cell carcinoma (SCC), and adjacent normal lung tissues (NC) were extracted from data obtained via a comprehensive gene expression analysis, and plotted. Horizontal bars indicate medians, ^*^P<0.0001. (B) FAM83B expression was examined by western blotting of samples from Ad (5 cases), SCC (5 cases), and NC (3 cases). GAPDH protein expression was examined as an internal control. (C) Immunohistochemistry (IHC) of FAM83B in Ad, SCC, and NC. Paraffin sections were stained using an anti-FAM83B antibody, which was detected by DAB staining. Representative images for each tissue are shown. Boxed regions in the left column are magnified and shown in the right column. Note that immunoreactivity for FAM83B was detected in the cytoplasm and near plasma membranes (arrowheads) in SCC, but were hardly detected in Ad and NC. Scale bars, 100 μm.

We first extracted the expression ratios of FAM83B from the comprehensive gene expression analysis data, and compared these among 20 cases of SCC, 60 cases of adenocarcinoma, and 64 samples of adjacent normal tissue. As shown in Fig. 1A, FAM83B mRNA levels in SCC were significantly higher than those in adenocarcinoma (P<0.0001) and normal tissues (P<0.0001), but there was no significant difference between levels in adenocarcinoma and normal tissues (Fig. 1A). We next analyzed FAM83B protein expression in five SCC cases, five adenocarcinoma cases, and three normal tissue samples by western blotting. FAM83B protein was detected as a signal with an apparent molecular mass of ~110 kDa. The FAM83B signal intensities in lung cancer were stronger than that in adjacent normal tissue, and the SCC samples expressed FAM83B at higher levels in comparison with the adenocarcinoma samples (Fig. 1B).

### Localization of FAM83B in lung cancer and normal lung tissue

To evaluate FAM83B expression in paraffin-embedded tissue samples that were retrospectively collected, we attempted immunohistochemical analyses using anti-FAM83B polyclonal antibodies. As shown in Fig. 1C, while the FAM83B signal was only weakly or barely detected in adjacent normal and lung adenocarcinoma tissues, some SCC tissues showed highly intense signals. The staining patterns of FAM83B differed in the same tissue sample as well as among SCC cases; it was often observed along the cell surface and occasionally in the cytoplasm, and in other cells it was localized both in the plasma membranes and cytoplasm (Fig. 1C). When positivity was examined, 94.7% of SCCs (107 out of 113 cases) and 14.7% of adenocarcinoma (15 out of 102 cases) were positive, and overall 56.7% (122 out of 215 cases) of lung cancers were positive. In contrast, all corresponding adjacent normal lung tissues were negative. Thus, sensitivity and specificity were calculated as 94.5 and 85.3%, respectively.

### Relationship between FAM83B and clinicopathological variables of SCC

**Figure 2:**
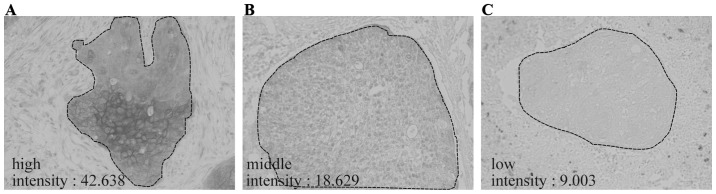

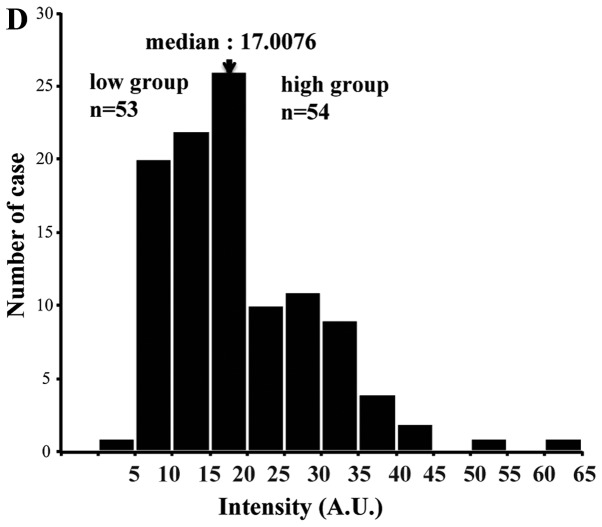

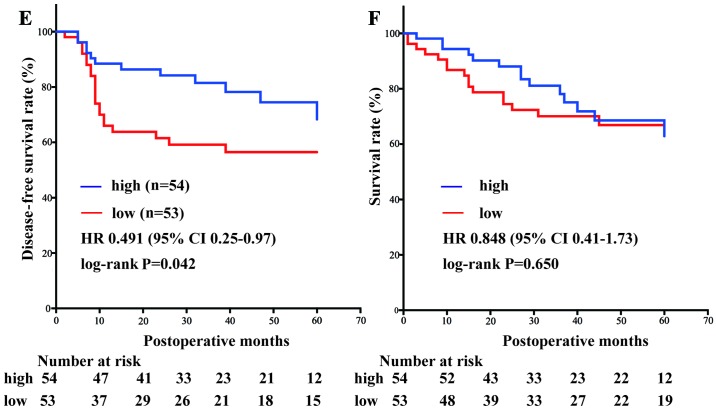
Immunohistochemical quantification of the family with sequence similarity 83 member B (FAM83B) expression and association with clinical outcomes among squamous cell carcinoma (SCC) patients. (A–D) Immunohistochemical images for FAM83B with (A) high, (B) medium, and (C) low intensities. During the imaging process, cancer regions (dotted line) were extracted and their average intensities were measured. (D) Values for 107 SCC clinical samples are shown as a histogram. Patients were divided in FAM83B-high and -low expression groups. Kaplan-Meier curves for (E) disease-free survival (DFS) and (F) overall survival (OS) in the high (blue line) and low (red line) expression groups. Statistical analysis was performed using the log-rank test.

The immunoreactivities for FAM83B varied among SCC tissues (Fig. 2A–C). Therefore, to examine the relationship between FAM83B protein levels and clinicopathological factors, FAM83B signal intensities were quantified by image analysis. When 107 FAM83B-positive cases of lung SCC were analyzed, the intensity values for FAM83B were distributed from 3.37 to 63.79 with a median of 17.01 (Fig. 2D). Then, the 107 cases were divided into a high-expression group (54 cases) and a low-expression group (53 cases), and were subjected to an association analysis with clinicopathological factors. Clinicopathological data for SCC patients included in this analysis are summarized in Table I. Notably, the majority of patients were male (88.8%), aged ≥65 years (81.3%), and smokers (Brinkman index ≥600, 86.0%). Percentages of patients with pathologic stage I and II+III were 57.0 and 43.0%, respectively. As a result, a marginal relationship between FAM83B and vascular invasion was found but this was not statistically significant (P=0.066), while there was no significant association between FAM83B protein expression and any of the factors examined including age, gender, smoking history, pT factor, pN factor, p stage, tumor differentiation, pleural invasion, or lymphatic vessel invasion.

### Survival outcomes according to FAM83B expression

We drew Kaplan-Meier survival estimates for the OS and DFS and compared the two groups using the log-rank test. In the high-expression group, there was a significant extension in the DFS (HR: 0.491, P=0.042; Fig. 2E) while OS did not differ significantly (HR: 0.848, P=0.650; Fig. 2F). We also applied univariate analysis to evaluate associations between DFS and several important clinicopathological factors including age, gender, smoking history, pT factor, pN factor, p stage, tumor differentiation, pleural invasion, lymphatic vessel invasion, vascular invasion, or FAM83B expression by the Cox proportional hazards model. pT factor, pN factor, p stage, pleural invasion, lymphatic vessel invasion, vascular invasion, and low protein expression of FAM83B were significantly associated with DFS (Table II). In a multivariate statistical analysis, however, the association did not reach statistical significance (P=0.197) (Table II), indicating that FAM83B expression could not be considered as an independent prognostic factor.

## Discussion

It has recently been reported that FAM83B can act as an important intermediary in aberrant EGFR/RAS signaling, and is actually highly expressed in several cancer tissues, such as breast and lung cancer (18). In the present study, we intensively examined the expression of FAM83B in lung cancer at both the mRNA and protein level, and found that it was highly expressed in lung SCC rather than in lung adenocarcinoma or adjacent non-cancer regions. Importantly, higher FAM83B expression evaluated by IHC was associated with longer DFS. Previously, Cipriano *et al* (18) investigated a microarray data set obtained from Oncomine (https://www.oncomine.org/), and found that FAM83B expression was associated with specific cancer subtypes, increased tumor grade, and decreased OS. In the case of lung cancers, they demonstrated that FAM83B expression was higher in SCC than adenocarcinoma (P=0.00084), and that it was associated with increasing T stage (P=0.016). Therefore, our data support their conclusions by additionally examining FAM83B protein levels, and further provide significant evidence that it is associated with better prognosis of SCC.

Lung cancer is divided into two major subgroups, small-cell lung cancer or NSCLC, by its clinical features and the selection of treatment type. Thus, in the past, NSCLC was treated according to a ‘uniform’ strategy. However, it has recently been recognized that histological subtypes are also important for lung cancer treatment, because several optional treatments have been proposed as specific for non-SCC. Unfortunately, there is no effective therapy for lung SCC. Therefore, accurate pathological diagnosis, particularly to discriminate non-SCC from SCC in biopsy samples, is an important step in its treatment. However, it has recently been reported that ~20% of hematoxylin-eosin-stained biopsy specimens from NSCLC fail to be appropriately diagnosed, and are known as ‘not otherwise specified’ with poor prognosis (22). Therefore, more effective diagnostic markers for each tissue type of NSCLC are required.

To date, only a few markers for lung SCC have been found, including cytokeratin 5/6 (CK5/6) and p63. CK5/6 is used as a marker of SCC, and its immunohistochemical detection showed 61–100% sensitivity and 79–93% specificity for SCC (23,24). p63 is a transcription factor belonging to the p53 family, and has been clinically used as a diagnostic marker (25). However, problems occasionally result because of its low specificity; it also shows positivity in 16–65% of lung adenocarcinomas (26). Recently, development of specific antibodies against p40 (ΔNp63) together with immunohistochemical evaluation of TTF-1 and p40 have made it possible to completely discriminate lung adenocarcinoma and SCC (27). Immunohistochemical detection of FAM83B in this study showed 94.5% sensitivity and 85.3% specificity for SCC. This highly accurate result indicates that FAM83B could be a reliable diagnostic marker for lung SCC. Further development of the detection system would be helpful for accurate and rapid diagnosis.

Prognosis of lung SCC is generally worse than that of lung adenocarcinoma (28). It is well known that lung SCC is associated with high smoking rates and complications such as interstitial pneumonia and chronic obstructive pulmonary disease resulting from smoking history, which hampers optimal treatments of chemotherapy, including adjuvant chemotherapy. Therefore, selection of appropriate treatments in so-called ‘personalized therapy’ is more important in determining the treatment strategy for lung SCC, where the information of prognostic biomarkers with higher reliability is helpful. Our data demonstrated that patients with high FAM83B expression tended to exhibit longer DFS (P=0.042), indicating that FAM83B is a candidate biomarker that can predict prognosis of SCC.

At present, we do not know the mechanism by which high expression of FAM83B results in longer DFS. FAM83B protein has an amino-terminal domain of unknown function (DUF1669), which is conserved among FAM83 members and contains a putative phospholipase D-like motif that is critical for FAM83B-mediated transformation activity. It has previously been shown that FAM83B can associate with CRAF, p85α and p110α subunits of PI3K, AKT, and EGFR (18,29,30), and is also able to activate phospholipase D via interaction with EGFR (29). These researchers concluded that FAM83B is involved downstream of EGFR, mediating both the MAPK and PI3K/AKT signaling pathways. Consequently, increased expression of FAM83B resulted in the transformation of human mammary epithelial cells (18,29,30). This mechanistic model is consistent with the fact that FAM83B is highly expressed in breast cancer, and that its expression level is associated with its malignancy (18). However, in the case of lung SCC, our results indicate that high expression of FAM83B would predict better prognosis. Although it seems contradictory, there has been another example. The transcription factor SOX2 has been identified as an amplified lineage-survival oncogene in lung and esophageal SCC, and its overexpression has been shown to be associated with better prognosis (31–33). The most plausible explanation would be that SOX2 expression might promote squamous differentiation rather than malignant dedifferentiation. FAM83B may also be involved in a similar mechanism, though further studies are required.

Proteome analyses have identified FAM83B as a novel interactor for APC and AXIN-1, both of which are components of a destruction complex of β-catenin and thus regulate the WNT signaling pathway (34). Such additional signaling pathways together with EGFR pathways may be differentially operated in different tissues and during the context of differentiation. Taken together, our findings suggest that although potential FAM83B-targeted therapy might be effective for breast cancer, it would not necessarily be true for lung SCC. More detailed analyses of fundamental signaling pathways using cell lines derived from SCC would give us better conclusions for the function of FAM83B in lung SCC.

## Figures and Tables

**Table I:** Relationship between FAM83B expression and clinicopathological parameters in SCC.

		FAM83B expression		
			
Characteristic	Total n=107	FAM83B high n=54 (50.5%)	FAM83B low n=53 (49.5%)	P: high vs. low
Gender				
Female	12 (11.2%)	7 (58.3%)	5 (41.7%)	0.563
Male	95 (88.8%)	47 (49.5%)	48 (50.5%)	
Age (years)				
<65	20 (18.7%)	7 (35.0%)	13 (65.0%)	0.125
≥65	87 (81.3%)	47 (54.0%)	40 (46.0%)	
Smoking history (BI)				
<600	15 (14.0%)	8 (53.3%)	7 (46.7%)	0.811
≥600	92 (86.0%)	46 (50.0%)	46 (50.0%)	
pT factor				
T1	36 (33.6%)	22 (61.1%)	14 (38.9%)	0.117
T2+T3	71 (66.4%)	32 (45.1%)	39 (54.9%)	
pN factor				
N0	76 (71.0%)	41 (53.9%)	35 (46.1%)	0.260
N1+N2	31 (29.0%)	13 (41.9%)	18 (58.1%)	
p-TNM stage				
Stage I	61 (57.0%)	33 (54.1%)	28 (45.9%)	0.387
Stage II/III	46 (43.0%)	21 (45.7%)	25 (54.3%)	
Tumor differentiation				
Well/moderate	80 (74.8%)	43 (53.8%)	37 (46.2%)	0.242
Poorly	27 (25.2%)	11 (40.7%)	16 (59.3%)	
Pleural invasion				
Positive	34 (31.8%)	14 (41.2%)	20 (58.8%)	0.190
Negative	73 (68.2%)	40 (54.8%)	33 (45.2%)	
Lymphatic vessel invasion				
Positive	46 (43.0%)	20 (43.5%)	26 (56.5%)	0.209
Negative	61 (57.0%)	34 (55.7%)	27 (44.3%)	
Vascular invasion				
Positive	53 (49.5%)	22 (41.5%)	31 (58.5%)	0.066
Negative	54 (50.5%)	32 (59.3%)	22 (40.7%)	

FAM83B, family with sequence similarity 83, member B; SCC, squamous cell carcinoma; BI, Brinkman index.

**Table II:** Cox proportional hazards model analysis of prognostic factors in patients with SCC.

Variables	Hazard ratio (95% Cl)	Unfavorable/favorable	P
Univariate analysis			
FAM83B	0.489 (0.240–0.996)	Weak/strong	0.049
Age (years)	1.088 (0.449–2.637)	≥65/<65	0.852
Gender	1.077 (0.379–3.063)	Male/female	0.889
pT factor	3.142 (1.293–7.638)	T2+T3/T1	0.012
pN factor	2.577 (1.288–5.156)	N1+N2/N0	0.007
p stage	2.309 (1.156–4.611)	II+III/I	0.018
Pleural invasion	4.167 (2.088–8.316)	Positive/negative	0.0001
Lymphatic vessel invasion	3.482 (1.703–7.122)	Positive/negative	0.001
Vascular invasion	2.302 (1.131–4.684)	Positive/negative	0.021
Multivariate analysis			
FAM83B	0.610 (0.288–1.294)	Weak/strong	0.197
Age (years)	1.321 (0.519–3.362)	≥65/<65	0.559
Gender	1.385 (0.467–4.108)	Male/female	0.557
pT factor	1.685 (0.557–5.096)	T2+T3/T1	0.355
pN factor	1.767 (0.577–5.408)	N1+N2/N0	0.319
p stage	0.960 (0.331–2.784)	II+III/I	0.94
Pleural invasion	2.538 (1.084–5.944)	Positive/negative	0.032
Lymphatic vessel invasion	2.453 (1.072–5.615)	Positive/negative	0.034
Vascular invasion	0.955 (0.423–2.157)	Positive/negative	0.912

SCC, squamous cell carcinoma; FAM83B, family with sequence similarity 83, member B.
